# Predicting Adolescent Intervention Non-responsiveness for Precision HIV Prevention Using Machine Learning

**DOI:** 10.1007/s10461-022-03874-4

**Published:** 2022-10-18

**Authors:** Bo Wang, Feifan Liu, Lynette Deveaux, Arlene Ash, Ben Gerber, Jeroan Allison, Carly Herbert, Maxwell Poitier, Karen MacDonell, Xiaoming Li, Bonita Stanton

**Affiliations:** 1 Department of Population and Quantitative Health Sciences, University of Massachusetts Chan Medical School, 368 Plantation Street, Albert Sherman Center, Worcester, MA 01605 USA; 2 Office of HIV/AIDS, Ministry of Health, Shirley Street, Nassau, Bahamas; 3 Department of Health Promotion, Education, and Behavior, University of South Carolina Arnold School of Public, Columbia, SC USA; 4 Department of Family Medicine and Public Health Sciences, Wayne State University School of Medicine, Detroit, MI USA; 5 Hackensack Meridian School of Medicine, 340 Kingsland ST., Nutley, NJ 07110 USA

**Keywords:** Machine learning, HIV prevention, Condom use skills, Self-efficacy, Intervention non-responsiveness, Prediction, Precision prevention

## Abstract

Interventions to teach protective behaviors may be differentially effective within an adolescent population. Identifying the characteristics of youth who are less likely to respond to an intervention can guide program modifications to improve its effectiveness. Using comprehensive longitudinal data on adolescent risk behaviors, perceptions, sensation-seeking, peer and family influence, and neighborhood risk factors from 2564 grade 10–12 students in The Bahamas, this study employs machine learning approaches (support vector machines, logistic regression, decision tree, and random forest) to identify important predictors of non-responsiveness for precision prevention. We used 80% of the data to train the models and the rest for model testing. Among different machine learning algorithms, the random forest model using longitudinal data and the Boruta feature selection approach predicted intervention non-responsiveness best, achieving sensitivity of 85.4%, specificity of 78.4% and AUROC of 0.93 on the training data, and sensitivity of 84.3%, specificity of 67.1%, and AUROC of 0.85 on the test data. Key predictors include self-efficacy, perceived response cost, parent monitoring, vulnerability, response efficacy, HIV/AIDS knowledge, communication about condom use, and severity of HIV/STI. Machine learning can yield powerful predictive models to identify adolescents who are unlikely to respond to an intervention. Such models can guide the development of alternative strategies that may be more effective with intervention non-responders.

**Supplementary Information:**

The online version contains supplementary material available at 10.1007/s10461-022-03874-4.

## Introduction

### Adolescent Sexual Risk Behaviors Remain High

Adolescence is characterized by significant physical and psychological development and is a prominent developmental period for experimentation and risk-taking [1]. Sexual risk behaviors, including early sexual debut, unprotected sex, and sex with multiple partners, place adolescents at high risk for negative health outcomes such as unintended pregnancy, sexually transmitted infections [STI], and HIV [2–5]. Of considerable concern, rates of HIV diagnoses have remained stable over the past 20 years for adolescents, despite decreasing substantially among all other age groups [6]. Many evidence-based HIV prevention interventions have been implemented in schools, communities, and clinic settings, with measurable effects on condom use, self-efficacy, and behaviors [7]. However, rates of adolescent sexual risk behaviors remain high. In 2019, only 54% of sexually active U.S. high school students reported using condoms in their most recent sexual encounter [8]. To optimize the health outcomes of adolescents and allocate resources to target this high-risk group for precision prevention efforts [9], it is important to understand why and for whom behavioral interventions are less effective.

### Intervention Programs are Differentially Effective for Youth

Several studies demonstrate differential effectiveness of various adolescent HIV risk reduction interventions [10, 11]. In one study, a sexual health intervention improved condom use intention among American Indian adolescent girls aged 13–15 years who had never had vaginal sex, but was less effective among boys, adolescents ages 16–19, and those already sexually active [11]. In a clinic-based condom use intervention among youth living with HIV found that the intervention was differentially effective across three sexual-risk trajectories [12]. While the intervention was effective in reducing unprotected sex among adolescents categorized in the “persistent low sexual risk” and “high and growing sexual risk” groups, the intervention was ineffective in a third group, that they labeled “delayed high sexual risk” [12].

Few studies have explored non-responsiveness to sexual-risk reduction interventions. Sales et al. studied non-responsiveness to a clinic-based STI/HIV behavioral intervention among African American adolescent and young adult women targeting condom use [13–15]. Non-responders more often reported partner or relationship-related issues (instability, infidelity), power dynamics, substance abuse, and a perceived inability to change their condom use [14]. In adjusted analyses, non-responders had higher levels of sensation seeking behaviors, a boyfriend at baseline, and a physical abuse history [13, 15]. Non-responders also showed no significant improvement in partner communication self-efficacy and reported more fear of condom negotiation [14]. Overall, limited research has been conducted on adolescent responsiveness to HIV prevention interventions. Identifying the characteristics of youth who are unresponsive to the interventions could be helpful in modifying programs to improve their effectiveness.

### Precision Prevention and Machine Learning for HIV Risk Prediction

Adolescent HIV risk involves complex behaviors influenced by individual, social, environmental, and personal factors and their interactions [16], that make some interventions more effective for some people than for others. Thus, there is growing interest in precision prevention, a term adapted from the discipline of precision medicine [17]; its goal is to tailor “the right intervention to the right population at the right time” [18]. Precision prevention requires understanding the diverse, individual needs within our population, that can inform the tailoring of appropriate supports and resources. With this framework, we explore complex longitudinal adolescent behavioral data to identify risk factors associated with non-response to a behavioral intervention. These data present numerous analytic challenges to classical statistical models, including nonlinear dependencies and unknown interactions that rarely conform to classical statistical assumptions [19]. Traditional statistical approaches to reduce analytic complexity can obscure important non-linearities or interactions [20]. In this context, various machine leaning (ML) algorithms have been identified as being potentially more effective for developing optimal prediction models. ML algorithms, especially supervised learning algorithms, seek models with optimal predictive performance based on pre-specified evaluation criteria [21]. ML approaches are well suited to data with multi-level potential predictors; its automated strategies (e.g., k-fold cross-validation) help guard against overfitting (i.e., building an excessively complicated model that performs poorly in new data) [22].

Existing studies have employed ML to build HIV prediction models to identify patients who are at risk for acquiring HIV and thus may benefit from HIV pre-exposure prophylaxis and to identify adolescents who are likely to engage in HIV risk behaviors (e.g., having multiple sex partners) [23–26]. ML has also been used to identify socio-behavioral predictors of HIV positivity [27], for predicting uptake of HIV testing among substance users [28], and for predicting early virological suppression in HIV patients [29]. In several studies, ML models have achieved better classification accuracy than logistic regression [25, 28]. However, most studies employing ML algorithms for prediction rely on cross-sectional data [30]. To our knowledge, there has been no study employed ML modeling to predict adolescent differential intervention response to behavioral interventions, particularly using rich longitudinal data.

### Theoretical Framework for Adolescent Risk Behavior

We follow Protection Motivation Theory (PMT), a social cognitive framework that has been used to investigate various risk and protective behaviors, including adolescent HIV risk behaviors [31–33]. PMT views the cognitive process of behavior change in terms of individual perceived threat and coping appraisal, helping to explain why people continue to engage in unhealthy behaviors, despite understanding the health risks [34]. A maladaptive response such as unprotected sex is mediated by a balance between perceived rewards (*intrinsic rewards*, e.g., favorable attitudes or feelings toward sex, and *extrinsic rewards*, e.g., peer influences) and perceived threat from participating in the maladaptive behavior (*perceived severity,* or negative consequences, such as pregnancy or STI/HIV) and *vulnerability* (likelihood of harm from those consequences). An adaptive response (such as using a condom) is mediated by balancing the *response efficacy* (the effectiveness of the protective behavior in lessening the health threat), *self-efficacy* (perceived ability to adopt the behavior), and the *response cost* (barriers or inconveniences) of performing the behavior. These two appraisal pathways combine to form *protection motivation* (intention) to respond to a potential threat in either an adaptive (protective behavior) or maladaptive (risk behavior) manner [34, 35].

### Aims of the Current Study

This study investigates the effectiveness of ML algorithms for predicting adolescent HIV intervention responsiveness. We use comprehensive, longitudinal data from the Bahamian Focus on Older Youth (“BFOOY”) intervention study, a large randomized controlled trial. Our ML models identify adolescents who are unlikely to respond to HIV prevention interventions. Specifically, in this study, we: (1) developed high-performance prediction models for adolescent intervention non-responders by applying ML techniques to complex longitudinal data; (2) compared prediction model variants to determine the most effective and parsimonious models for HIV intervention non-responsiveness; and (3) identified the factors contributing most to HIV intervention non-responsiveness. This will enable HIV “precision prevention” efforts, targeting high-risk adolescent subgroups.

## Materials and Methods

### Bahamian Focus on Older Youth (“BFOOY”) Study and Data

The data for this study were collected as part of a randomized, controlled trial of a theory-based, HIV prevention intervention: Bahamian Focus on Older Youth (“BFOOY”) [36]. This study involved youth and their parents from 146 grade-10 Health and Family Life Education (HFLE) classrooms in all eight government high schools on the most populated Bahamian island.

BFOOY is an age-appropriate 8-session behavioral program, which is an evidence-based, life skills curriculum designed to reduce risk-taking behaviors related to HIV/STI transmission and teen pregnancy. There were two versions of intervention for parents: a parental monitoring intervention entitled “Caribbean Informed Parents and Children Together” (CImPACT) and a goal-setting intervention (Goal for It [GFI]). CImPACT is a single session intervention including a 22-min educational video filmed in The Bahamas which focuses on effective parent-adolescent communication and listening strategies related to difficult topics and safe-sex followed by two role-plays for the parent and youth, a discussion, and a condom demonstration. GFI is a 22-min video describing the process of career development, followed by a parent–child question and answer period. The control condition was the existing Bahamian Health and Family Life Education (HFLE) curriculum, which focuses on a range of health topics, including a factual presentation of HIV and pregnancy prevention and discussions of marriage and parenting. The BFOOY + CImAPCT intervention is a 9-session behavioral intervention.

A total of 2564 grade-10 youths were enrolled and followed for 24 months over 2008–2011. These students were randomly assigned at the level of the classroom to one of four intervention conditions: BFOOY + CImPACT, BFOOY + GFI, BFOOY only, or HFLE only (see detailed description [36]). The core BFOOY intervention was offered with and without each of two intervention versions for parents: CImPACT and GFI. Outcomes were assessed on the entire sample at 6, 12, 18, and 24 months post-intervention. Follow-up rates were high: 84%, 79%, 78%, 78% at 6, 12, 18 and 24 months, respectively. The mean age of youth at baseline was 14.5 years (range 13 to 17 years). Ninety-nine percent of youth were of African descent.

Participants completed a self-administered questionnaire, requiring ~ 45 min. Detailed information was collected with each survey on adolescent risk behaviors, neighborhood risk, protection motivation theory (PMT) perceptions, sensation-seeking, perceived peer risk involvement, parental monitoring, parent-youth communications, depression, behavioral intentions, condom use skills and HIV/AIDS knowledge with > 50 high-level features and 200 items. (See Supplement Table 1 for assessment features and targets).

### Study Outcomes

Intervention non-responsiveness. We predicted non-responsiveness (NR) to HIV prevention intervention using two definitions: (1) non-responsiveness based on condom use skills (NR-Skill): if the condom use skill score is not improved at time 5 (24 months post-intervention) compared to baseline (Time 1, intervention start), the value is set as 1, and otherwise, 0; and (2) non-responsiveness based on self-efficacy (NR-Efficacy): if the self-efficacy score is not improved at time 5 compared to baseline, the value is set as 1, otherwise is set as 0. For the few cases (2%) where time 5 scores were not available, we used time 4 scores.

Condom-use skills. The Condom-Use Skills Checklist [37] assessed understanding of correct condom use. From among 16 items, students identified the 8 correct steps. We assigned 1 point for each item that was correctly marked (0 = incorrect; a = 0.66), resulting in a summary score of 1 to 16 for each participant.

Condom-use self-efficacy. We assessed a youth’s self-perceived ability to perform tasks to achieve protection through condom use through 5 items (e.g., “I could put on a condom correctly”). We measured each response on a 5-point Likert scale (1 = strongly disagree; 5 = strongly agree; a = 0.81). A composite score was calculated as a mean score across the 5 items (range 1–5).

### Machine Learning for Non-responsiveness Prediction

**Fig. 1:**
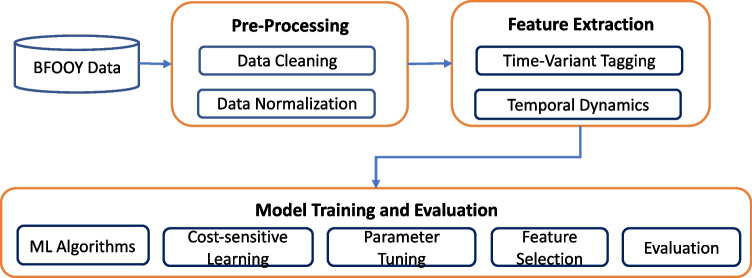
Machine learning workflow for non-responsiveness prediction

We formulated the non-responsiveness prediction task as a binary classification problem. For each student, the trained model was used to predict whether he/she is non-responsive to the HIV prevention intervention (NR-Skill = 1 or NR-Efficacy = 1) using longitudinal data collected at times 1–4. Study data were pre-processed, features were extracted, and then models were trained and evaluated (Fig. 1).

#### Data Pre-processing

##### Data Cleaning

We removed 276 participants from among the total number of participants (n = 2564) due to missing values on either of the two outcomes (NR-Skill, NR-Efficacy). We also removed participants assigned to the control group (n = 772), as they could not contribute information regarding an intervention that they did not receive. We aggregated the values for each subscale (e.g., PMT constructs) into a single feature rather than including item-level responses. We explicitly encoded all other missing values as “-1”, an additional feature dimension to be leveraged in model training, which has been shown effective on previous studies [38].

##### Data Normalization

Different features (predictors) take different value ranges. To avoid the potential dominant influence by variables with extreme values on the model training process, we applied the common “min–max normalization” to code every feature on the same range of [0.0, 1.0].

#### Feature Extraction

An observation in our dataset represents an individual student at a single point in time, which is represented as a list (feature vector) of values each corresponding one predictor (feature). Data for the *m*th student at time *t* is coded as: *x*_*m*_^*t*^ = (*x*_*m1*_^*t*^, *x*_*m2*_^*t*^, …, *x*_*mn*_^*t*^), where *x*_*m*_^*t*^ is the feature vector, and *x*_*mn*_^*t*^ indicates the feature value for the *n*^*th*^ predictor (feature) measured at time point *t* for person *m*. Our goal is to leverage the longitudinal information collected at times 1–4 to predict non-responsiveness at the end of the HIV prevention intervention; given a feature vector *x*_*m*_^*t*^, that encodes longitudinal information collected at time points 1–4, the system will predict whether the student is responsive to the intervention at time 5.

To make good use of our longitudinal data, we used three types of features in building the classification model. As we explain in the next two paragraphs, the classification model includes time-variant tagging features as well as temporal dynamic features. The latter consists of two subgroups: sliding windows and derivative dynamic features.

##### Time-Variant Tagging Features

The same predictor is attached with different identifiers to distinguish values collected at different times (e.g., Self_efficacy_3 means the Self_efficacy score collected at Time 3).

##### Temporal Dynamic Features

- Sliding windows (SW) features: a sliding window (time interval) is characterized by a window size (length in follow-up) and the sliding step size (time points). It can capture the localized dynamics of the data sequence aligned with the window. When the window slides from left to right (one step at a time) until it reaches the end of the sequence (last follow-up), it can capture overlapping dynamic characteristics through the whole sequence. We applied sliding windows of multiple lengths with the sliding step size set as 1. We calculated descriptive statistics as features, including the mean and standard deviation values of a particular feature within each sliding window. Those features are distinguished by attaching an affix to their names to indicate the corresponding time span of each sliding window. For instance, the string “SW_STD_13” affixed in the feature name indicates the standard deviation (STD) within a sliding window covering Times 1 through 3.
- Derivative dynamic (DN) features: DN features capture incremental (decremental) changes in the value of an attribute between adjacent time points. By comparing values of adjacent data points in the longitudinal sequence, local dynamic changes are captured for each attribute. For example, Self_efficacy_DN_34 represents the changes of Self_efficacy score between Time 3 and 4. Dynamic features have been found to be helpful in multivariate time series classification [39].

#### Model Training and Evaluation

*Machine Learning (ML) Algorithms* We explored four classic machine learning algorithms for predicting HIV-related risk behaviors: Support Vector Machines (SVM); Logistic regression (LG); Decision Tree (DT); and, Random forests (RF). These algorithms are widely used for classification problems, and each has its unique features and advantages. SVM employs the “max-margin principle”, to find a classification boundary such that the data points (from the responders/non-responders classes) which are the nearest to the boundary can both be separated but also maintain the largest possible margin to the boundary. The max-margin design of SVM typically leads to good system generalizability. LG is a simple linear classifier which works by taking a linear combination of features and applying non-linear sigmoid function to it. DT learns a tree structure hierarchically (through iterative splitting) with the goal of producing the most homogeneous groups; this is easy to visualize and interpret. RF is an ensemble version of DT by aggregating predictions from multiple decision trees for a better model, which is more robust against overfitting. Our analysis focused on building longitudinal prediction models using classic machine learning algorithms. To increase the applicability of the model on data without longitudinal monitoring, we also built and evaluated cross-sectional prediction models (including baseline factors only). In addition, our machine learning models accounted for intervention group assignment and the receipt of Focus on Youth in the Caribbean'' (FOYC) intervention in grade 6.

*Cost-sensitive Learning* We used cost-sensitive learning [40] to deal with the data imbalance between different categories (e.g., Non-responsiveness vs. Responsiveness). Specifically, the minority category (the one with fewer observations, in this case “non-responsiveness”) was assigned a larger weight during model training to mitigate the overwhelming influence of the majority category. It has demonstrated better performance than up-sampling and down-sampling approaches in our previous research [41].

*Parameter Tuning* We employed grid search to choose optimal parameters in the tenfold cross validation setting for each ML algorithm using the training data. The best parameter setting was further validated in the withheld test data.

*Feature Selection* Feature selection helps the model to identify informative variables and eliminate ones that are largely redundant, leading to better performance and generalizability. We explore four feature selection approaches: (1) Mutual Information (MI) [42], a filter method to select a subset of features based on dependencies among the features and target category labels; (2) LASSO [43], an embedded method for feature selection and regularization during the model training process; (3) Recursive Feature Elimination (RFE) [44], a “wrapper” method (evaluates on an external machine learning algorithm to identify optimal features) to select features by recursively considering smaller and smaller sets of features, based on the weights assigned to features by an external estimator; and (4) Boruta [45], another wrapper method with “all relevant” criteria instead of “minimal optimal”, which iteratively removes features that are statistically less relevant to the target outcome variable than a random probe.

*Evaluation Metrics* We used the following evaluation metrics: sensitivity, specificity, the area under the receiver operating characteristic curve (AUROC), and the area under the precision recall curve (AUPRC), which were reported on both training data (tenfold cross validation) and testing data.

*Experiment Setup and System Development* We employed the Scikit-Learn (version 0.24.2) [46] toolkit to develop our models. The linear kernel (a dot product of two feature vectors in a linearly separable space) was used for SVM in the study. We randomly sampled 80% of the data for training, holding out the rest for testing.

## Results

### Non-responsiveness Statistics

After data cleaning, there are 1495 and 1514 participants included for NR-Skill and NR-Efficacy analysis, respectively. BFOOY dataset demonstrated a good representation of both intervention responders (NR-skill 60%; NR-efficacy 59%) and non-responders NR-skill 40%; NR-Efficacy 41%) (supplement Table 2).

### Comparative Results Using Four Machine Learning Algorithms

tenfold cross validation (CV) results on the training data from 4 algorithms are shown in Table 1, where RF performed best for both NR-Skill and NR-Efficacy across most evaluation metrics, yielding AUROC of 0.842 and 0.928, AUPRC of 0.795 and 0.923 respectively. LG obtained the best sensitivity of 83.19% for NR-Skill, and DT achieved the best specificity of 84.37% for NR-Efficacy.

**Table 1:** Predictive performance of four machine learning models (tenfold CV prediction on training data)

	NR-Skill				NR-Efficacy			
	Sens (%)	Spec (%)	AUROC	AUPRC	Sens (%)	Spec (%)	AUROC	AUPRC
SVM	81.06	65.17	0.812	0.749	84.62	78.55	0.897	0.862
LG	**83.19**	60.07	0.811	0.747	83.83	79.83	0.901	0.879
DT	82.13	66.42	0.805	0.760	82.24	**84.37**	0.908	0.912
RF	82.77	**67.40**	**0.842**	**0.795**	**85.40**	78.41	**0.928**	**0.923**

Bold indicates the best value per evaluation metric

*SVM* support vector machines; *LG* logistic regression; *DT* decision trees; *RF* random forests; *Sens* sensitivity; *Spec* specificity; *AUROC* area under the ROC curve; *AUPRC* area under the precision-recall curve

### Effects of Longitudinal Modeling (RF)

To better understand how longitudinal data contribute to predictive accuracy, we developed a “RF-Base” system which only used the baseline information at time 1. The impacts of using longitudinal data is shown in Table 2. “RF” represents the system using longitudinal data (the same as the last row in Table 1). For both outcome definitions, RF outperforms RF-Base with a large margin (AUROC of 0.842 vs. 0.783 for NR-Skill, 0.928 vs. 0.901 for NR-Efficacy), despite slight lower performance on sensitivity.

**Table 2:** Effects of longitudinal modeling (tenfold CV prediction on training data)

	NR-Skill				NR-Efficacy			
	Sens (%)	Spec (%)	AUROC	AUPRC	Sens (%)	Spec (%)	AUROC	AUPRC
RF	82.77	**67.40**	**0.842**	**0.795**	85.40	**78.41**	**0.928**	**0.923**
RF-Base	**84.47**	52.66	0.783	0.716	**86.20**	72.29	0.901	0.898

Bold indicates the best value per evaluation metric

*RF* random forests; *RF-Base* random forests using baseline features only; *Sens* sensitivity; *Spec* specificity; *AUROC* area under the ROC curve; *AUPRC* area under the precision-recall curve

### Results on Feature Selection

Supplement Table 3 presents results for 4 different feature selection approaches (random forests as the final classifier). It shows that with less than half of the features, LASSO and RFE performs better than or equal to using all the features. Boruta can achieve comparable performance with less than 20 and 40 features (compared with using all the 677 and 702 features in the “No-FS” settings).

**Table 3:** Prediction performance on the test data using different feature selection methods

	NR-Skill				NR-Efficacy			
	Sens (%)	Spec (%)	AUROC	AUPRC	Sens (%)	Spec (%)	AUROC	AUPRC
MI	82.68	**69.19**	0.831	0.785	**81.74**	**79.79**	0.905	**0.899**
LASSO	82.68	66.86	**0.845**	**0.814**	80.87	78.72	**0.906**	0.898
RFE	82.68	66.86	0.843	0.813	**81.74**	**79.79**	0.903	0.893
Boruta	**84.25**	66.86	**0.845**	**0.817**	80.87	76.06	0.879	0.869
No_FS	82.68	67.44	0.843	0.804	80.87	80.85	0.905	0.897

Bold indicates the best value per evaluation metric

*Note* number of features selected by each method was optimized on the training data

*MI* mutual information; *LASSO* least absolute shrinkage and selection operator; *RFE* recursive feature elimination

### Performance on the Withheld Test Data

We applied the random forests (RF) models with different feature selection methods on the withheld test data (Table 3). Every feature selection approach showed very good generalizability; Boruta achieved the best sensitivity (84.25%) and AUROC (0.845) for predicting NR-Skill on the test data with less than 20 features.

### Model Interpretation

**Fig. 2:**
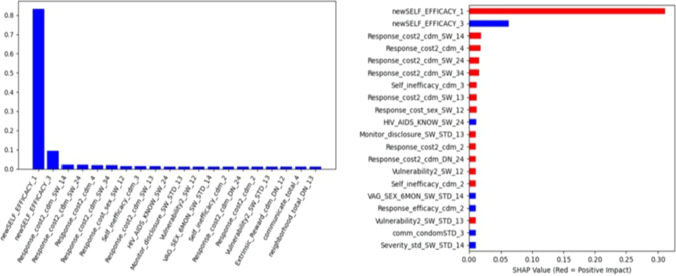
Top 20 features identified by the machine learning model

We extracted the 20 most important features learned by the machine learning model using two approaches: the model coefficient value (left) and the SHAP value (right) (Fig. 2). Among those top 20 features, 17 of them are shared by both of the approaches, despite different ranking order. The model coefficient value captures “Extrinsic_reward_cdm_DN_12”, “communicate_total_4”, “neighborhood_total_DN_13” while the SHAP value captures “Response_efficacy_cdm_2”, “comm_condomSTD_3”, “Severity_std_SW_STD_14”.

**Fig. 3:**
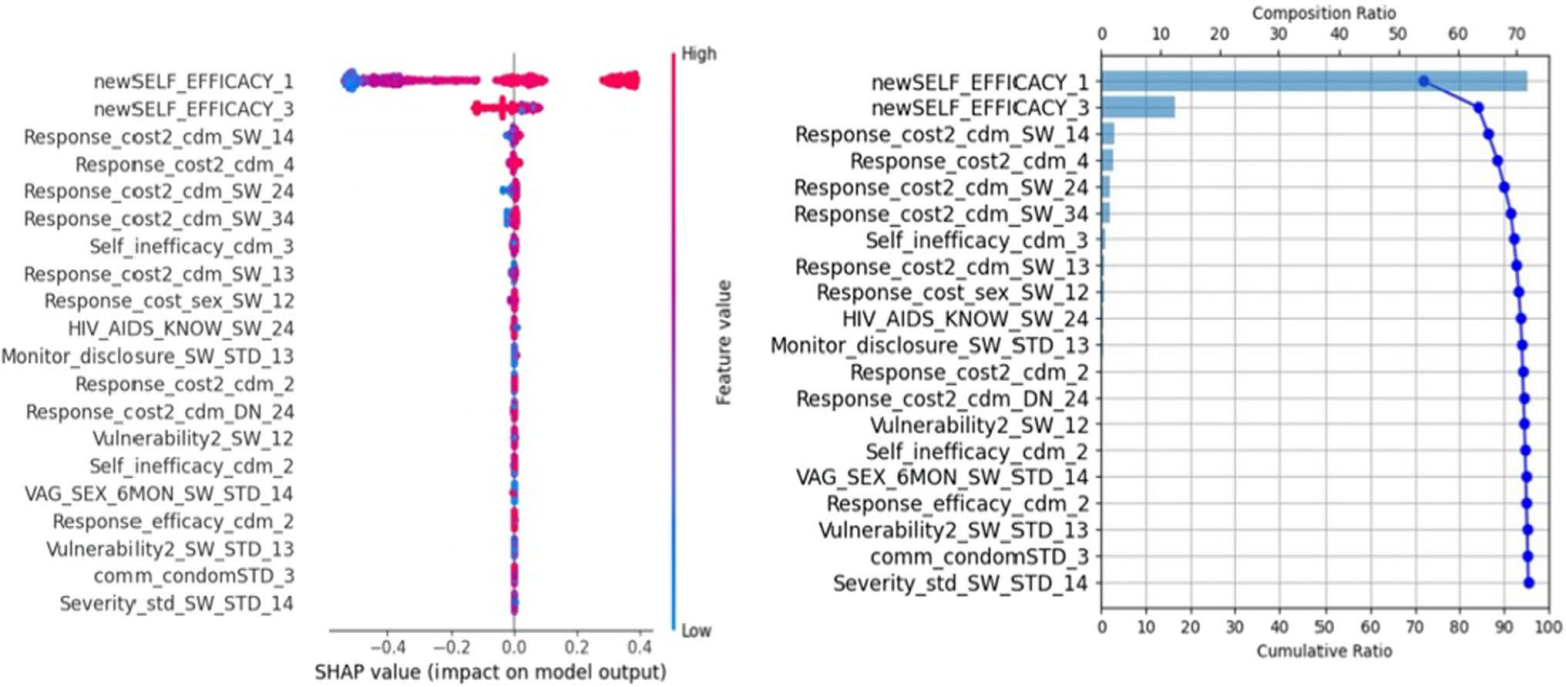
Impact of Top 20 features on model’s decision

Figure 3 shows the summary plot (left) and aggregated waterfall plot (right) on the training data based on SHAP values. The summary plot indicates positive and negative relationships of the top 20 predictors with the target variable, and each dot represent one individual sample in the training data. Red dots on the right side or blue dots on the left side indicate positive correlations. The deviation from the zero SHAP value indicates the larger impact in model decision making. It demonstrates that “SELF_EFFICACY” is the most important feature followed by “Response_cost” and “HIV_AIDS_KNOW” (refer to Discussion section for more details). The aggregated waterfall plot shows the accumulative interpretation of the model with top 20 important features, which shows those 20 features contribute more than 90% of the model reasoning process.

**Fig. 4:**
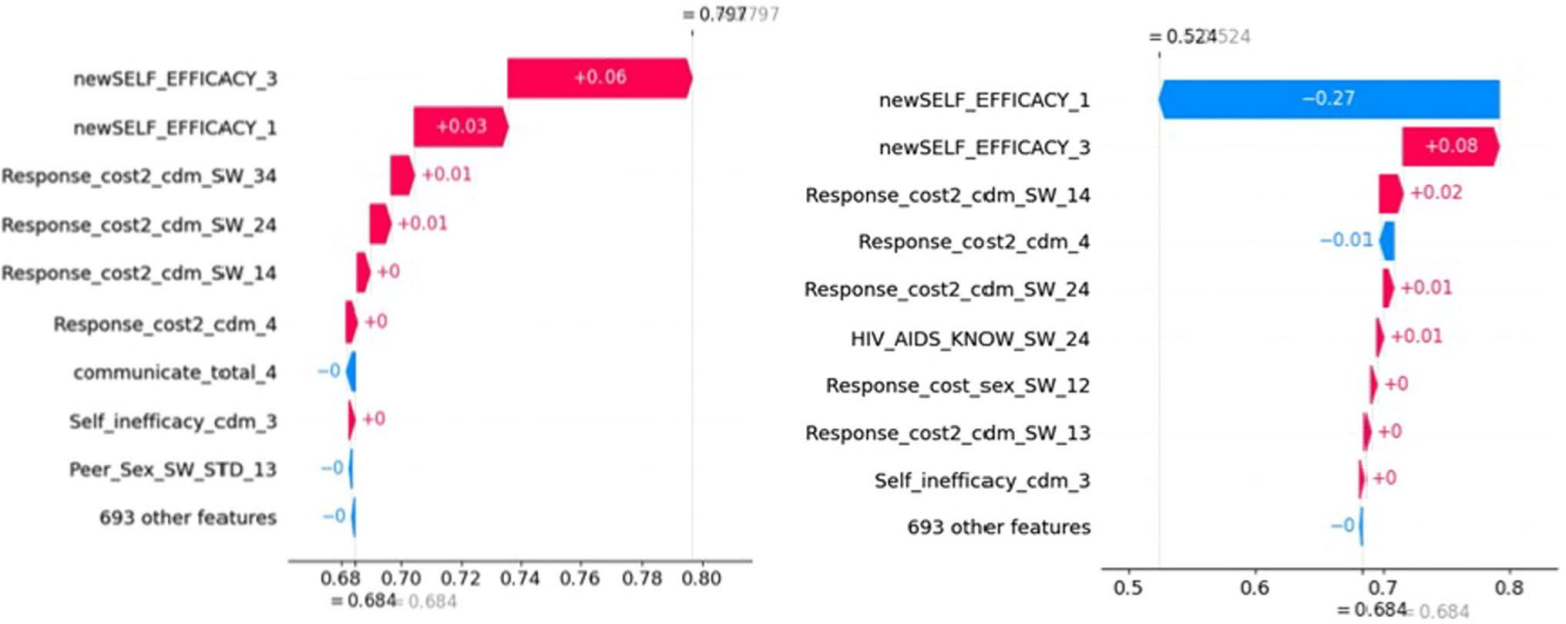
Waterfall plots to explain model’s decision-making process for two individual participants

In addition to providing global interpretation of the trained model, we used individual water plots to explain the decision-making process for each individual student on the testing data as shown in Fig. 4. It shows why a case receives its prediction impacted by different predictors. It starts with the bottom of a waterfall plot and adds (red) or subtracts (blue) the values to get to the final prediction. We can see that the same predictor (newSELF_EFFICACY_1) can affect the decision positively (left plot) or negatively (right plot). Also, for each individual, the top contributing predictors may be different.

## Discussion

To our knowledge, this is the first study to explore different machine learning algorithms in predicting adolescent non-responsiveness to an evidence-based HIV intervention using comprehensive longitudinal data. Four supervised machine learning models were developed, and their performance compared. Although all four of the models had fairly high accuracy, the random forest (RF) model showed the highest overall performance for predicting intervention non-responsiveness. The RF model also showed high predictive accuracy on the withheld testing data, indicating good generalizability with future similar samples. Our approach allowed us to identify several significant predictor variables for intervention non-responsiveness.

Our dataset had a good representation of both intervention responders (~ 60%) and non-responders (~ 40%). We compared four machine learning models for their predictive performance with hyperparameter optimization. Our experiments show that Random Forest consistently outperforms other three models, yielding a AUROC of 0.93 for NR-Efficacy and 0.84 for NR-Skill in the cross-validation setting on the training dataset, with good sensitivity of 83% and 85% respectively. Other machine learning models involving trees and regression algorithms performed with an average AUROC of 0.90 (NR-Efficacy) and 0.81 (NRI-Skill). Our experiments showed that longitudinal characteristics played an important role in improving the system’s prediction performance based on both AUROC and AUPRC. We tried different feature selection approaches to identify salient features before model training, and it was observed that with less but more important features, the system can achieve comparable or better performance on both training data and unseen test data. This shows that it is feasible to identify a limited number of features that are robustly associated with adolescent intervention responsiveness which can facilitate model interpretation and inform effective intervention implementation.

The many covariates investigated for the prediction models represented different domains including socio-demographics, various types of risk behaviors, neighborhood risk, peer influence, parental monitoring and communication, depression, and PMT constructs. Among the seven PMT constructs, self-efficacy and response cost are the most important predictors for intervention non-responsiveness. Youth who reported high level of self-efficacy at baseline are more likely to be intervention “non-responders” due to the ceiling effect [47], because youth with high initial scores on self-efficacy have little or no improvements in self-efficacy or condom use skills which are highly correlated with each other. Response cost is positively associated with intervention non-responsiveness, because adolescents’ perception of barriers to condom use (inconvenience, reduced sexual pleasure) is negatively related to condom use skills and self-efficacy [48]. On the other hand, response efficacy is negatively associated with intervention non-responsiveness [49]. Increases in vulnerability and parental monitoring are positively associated with intervention non-responsiveness. This may be because parents increase their monitoring efforts due to their children’s engagement in sexual risk behaviors (unprotected sex).

Although using longitudinal data improved predictive performance, it may not be available as it is hard to collect. To increase the applicability of the model on data without longitudinal monitoring, we evaluated cross-sectional prediction models. The RF model based on baseline data exhibited promising prediction accuracy, although it underperforms the RF model developed that used data collected after longitudinally. This finding is important because it suggests that public health research can apply machine learning methods on baseline data to identify “high-risk” adolescents who are likely to engage in HIV risk behaviors or who are unlikely to respond to the intervention. As data continue to accumulate (more waves of longitudinal data), the prediction model can be renewed using longitudinal data to improve its prediction accuracy. As efficient machine learning algorithms can help identify adolescents who are less responsive to the interventions in real time, an intensive and targeted intervention can be developed and delivered to them. Dissemination efforts targeting subgroups of youth with characteristics associated with non-responsiveness to a specific intervention could enhance the program’s impact.

This study has some limitations. First, the data used in this study are from Bahamian high school students (grade 10–12 youth). Although our model demonstrated good generalizability within this population, external evaluations are warranted to further understand how the ML algorithms and important predictors generalize to other adolescent populations. Second, we did not compare our ML prediction models with a sophisticated traditional statistical approach. This is because there are complex interactions among individual, peer, family and neighborhood risk factors, and collinearities and nonlinear relationships among these factors in our rich data. Traditional analytic methods are ill-equipped to analyze such complex data. Further, several studies showing that ML models result in higher classification accuracy than logistic regression have already been conducted [30, 33]. Third, there are approximately 40% non-responders, which include 17.2% of “inconsistent responders” whose scores increased but then relapsed by the end of follow-up period. In this study we combined non-responders and inconsistent responders to create a relatively balanced data for machine learning modeling. We will explore alternative definitions of non-responsiveness to capture short-term responders in the future work.

## Conclusions

Our study addressed an understudied area, the intersection of machine learning and HIV prevention. Different machine learning models have been developed to predict intervention non-responsiveness. Our study demonstrates that machine learning can yield powerful predictive models to identify adolescents who are unlikely to respond to an intervention, which establishes a foundation for future efforts to develop alternative targeted interventions for high-risk adolescents (precision HIV prevention). Machine learning HIV prediction has the potential to inform significant improvements in HIV prevention. Our framework provides a methodologic basis for harnessing the predictive power of machine learning for HIV prevention.

## Supplementary Information

Below is the link to the electronic supplementary material.

Supplementary file1 (DOCX 19 KB)

## Data Availability

Data and materials are available for reviewers upon request.
