# Supplementary material for: Predicting Adolescent Intervention Non-responsiveness for Precision HIV Prevention Using Machine Learning
Source: AIDS Behav. 2022 Oct 18;27(5):1392–402. doi: 10.1007/s10461-022-03874-4 (PMC10129965; doi:10.1007/s10461-022-03874-4)
Supplement: Supplementary file 1 — Supplementary file1 (DOCX 19 KB) [file 10461_2022_3874_MOESM1_ESM.docx]

| **Supplement Table 1 High-level features and targets from the BFOOY longitudinal study** | |
| --- | --- |
| Target outcomes | 1) having multiple sexual partners; 2) ever had sex |
| Features |  |
| Adolescent risk behaviors | Delinquent behaviors (7 items); substance use behaviors (8 items); sexual behaviors (14 items) |
| Neighborhood risk | Perceptions of alcohol use (2 items), marijuana or crack cocaine use (4 items), pushing or carrying drugs (2 items) among relatives and neighbors |
| PMT perceptions       (sexual initiation) | Extrinsic reward (5 items); intrinsic reward (1 item); perceived severity (3 items); vulnerability (3 items), self-efficacy (4 items); response efficacy (3 items); response cost (4 items) |
| PMT perceptions       (condom use) | Extrinsic reward (1 item); intrinsic reward (1 item); perceived severity (3 items); vulnerability (3 items), self-efficacy (6 items); response efficacy (3 items); response cost (6 items) |
| Peer risk behaviors | Sexual behavior (3 items); condom use (3 items); substance and drug use (4 items) |
| Parental monitoring | Parental knowledge (2 items), parental solicitation (1 item), parental control (1 item) and youth disclosure (3 items) |
| Parent-youth communication | Open communication (10 items); problematic communication (10 items) |
| Depression | Children’s Depression Inventory (10 items) |
| Values | Benevolence (4 items); universalism (6 items); self-direction (4 items); stimulation (3 items); hedonism (3 items); achievement (3 items); power (3 items); security (5 items); conformity (4 items); tradition (4 items) |
| Condom use skills | Correct statements (8 items); incorrect items (9 items) |
| Intentions | Drug use (3 items); having sex (1 item); pregnancy involvement (1 item); condom use (1 item); alcohol use (1 item); HIV/STD infection (2 items) |
| HIV/AIDS knowledge | AIDS transmission knowledge (8 items); prevention knowledge (8 items) |

| **Supplement Table 2**. BFOOY data statistics by two responsiveness outcomes | | |
| --- | --- | --- |
|  | Non-responsiveness | Responsiveness |
| NR-Skill | 597 (39.9%) | 898 (60.1%) |
| NR-Efficacy | 622 (41.1%) | 892 (58.9%) |

| **Supplement Table 3**. Prediction performance using different feature selection methods (10-fold CV prediction on training data) | | | | | | | | |
| --- | --- | --- | --- | --- | --- | --- | --- | --- |
|  | NR-Skill | | | | NR-Efficacy | | | |
|  | Sens | Spec | AUROC | AUPRC | Sens | Spec | AUROC | AUPRC |
| MI (600^*^, 400^**^) | 82.98% | 66.98% | 0.837 | 0.790 | **87.19%** | 77.99% | 0.929 | 0.924 |
| LASSO (300^*^,100^**^) | **83.19%** | **67.67%** | **0.844** | 0.798 | 85.00% | 78.39% | **0.930** | **0.925** |
| RFE (200^*^, 100^**^) | **83.19%** | 67.26% | 0.842 | **0.799** | 85.60% | **79.11%** | 0.929 | 0.923 |
| Boruta (<20^*^, <40^**^) | **83.19%** | 67.12% | 0.834 | 0.773 | 86.60% | 74.58% | 0.916 | 0.910 |
| No_FS (677^*^, 702^**^) | 82.77% | 67.40% | 0.842 | 0.795 | 85.40% | 78.41% | 0.928 | 0.923 |
| ^*^ Number of features selected when predicting NR-Skill; ^**^ indicates the number of features selected when predicting NR-Efficacy. | | | | | | | | |
